# Chronic Migraine Preventive Treatment by Prefrontal–Occipital Transcranial Direct Current Stimulation (tDCS): A Proof-of-Concept Study on the Effect of Psychiatric Comorbidities

**DOI:** 10.3389/fneur.2021.654900

**Published:** 2021-05-13

**Authors:** Giulio Mastria, Alessandro Viganò, Alessandra Corrado, Valentina Mancini, Cristina Pirillo, Simone Badini, Barbara Petolicchio, Massimiliano Toscano, Marta Altieri, Roberto Delle Chiaie, Vittorio Di Piero

**Affiliations:** ^1^Department of Human Neurosciences, Sapienza—University of Rome, Rome, Italy; ^2^My Space Lab, Department of Clinical Neuroscience, Centre Hospitalier Universitaire Vaudois (CHUV), University of Lausanne, Lausanne, Switzerland; ^3^Fondazione Don Carlo Gnocchi Onlus (IRCCS), Milan, Italy; ^4^Developmental Imaging and Psychopathology Laboratory, University of Geneva School of Medicine, Geneva, Switzerland; ^5^Department of Neurology—Fatebenefratelli Hospital, Rome, Italy; ^6^University Consortium for Adaptive Disorders and Head Pain—UCADH, Pavia, Italy

**Keywords:** tDCS, migraine, bipolar disorder, cerebellar stimulation, occipital cortex stimulation, dorsolateral prefrontal cortex stimulation, migraine overuse headache

## Abstract

Chronic migraine (CM) is often complicated by medication overuse headache (MOH) and psychiatric comorbidities that may influence the clinical outcome. This study aimed to investigate the relationship between psychiatric comorbidities and the effect of transcranial direct current stimulation (tDCS) in patients with CM with or without MOH. We recruited 16 consecutive CM patients who had an unsatisfactory response to at least three pharmacological preventive therapies. They were treated with anodal right-prefrontal and cathodal occipital tDCS (intensity: 2 mA, time: 20 min) three times per week for 4 weeks. All patients underwent a psychopathological assessment before and after treatment, and five of them were diagnosed with bipolar disorder (BD). After treatment, all the patients showed a significant decrease of severe and overall headache days per month. Despite having a higher migraine burden at baseline, patients with CM and BD showed a significantly greater reduction of severe headaches and psychiatric symptoms. Overall, tDCS seems to be effective in the treatment of CM patients with a poor response to different classes of pharmacological therapies, whereas BD status positively influences the response of migraineurs to tDCS.

## Introduction

Chronic migraine (CM) is a severe condition characterized by more than 15 headache days per month, 8 of which presenting migraine features, lasting at least 3 months (1). It represents the most common type of headache referring to specialized headache centers. CM is often irresponsive to medical treatment, especially when complicated by medication overuse headache (MOH) (ICHD-III 8.2) (2). Treatment adherence is quite poor and heavily affected by side effects (3, 4). Therefore, there is an increasing interest in therapeutic alternatives with fewer side effects, including non-pharmaceutical treatments. The poor outcome of preventive treatments is mostly attributable to CM multifactorial pathophysiology, in which electrophysiological alterations, psychiatric comorbidities, and environmental factors concur to reverberate pain (2).

Non-invasive neurostimulation through transcranial direct current stimulation (tDCS) has recently been suggested as a potentially useful tool for migraine prevention (5). In a simplified view, when tDCS is applied with two electrodes, one serving as the anode and the other as a cathode, positive stimulation increases neuronal excitability, whereas negative stimulation decreases it in most of the settings (6). TDCS may provide a safe and effective treatment, given the absence of severe side effects, generally occurring during the stimulation and rarely outlasting it (7). Moreover, tDCS may influence several areas of the brain according to the chosen montage (8), modifying targeted mechanisms of migraine (9) and therefore allowing a tailored intervention on each patient's pathophysiological profile.

Psychiatric comorbidities are frequently associated with CM, with or without MOH. Depression and anxiety are particularly common among these patients (10), as well as bipolar disorder (BD), both in its full-blown presentations and in the milder subthreshold forms of the bipolar spectrum, such as cyclothymia or type A behavioral pattern (11, 12).

The presence of such comorbidities has a detrimental impact on treatment outcome (13), being associated with a worse clinical condition, development of MOH, and reduced efficacy of pharmacological preventive therapies (14). TDCS has been applied to treat psychiatric diseases (15); therefore, it is reasonable to expect that specific montages might be effective in the treatment of both migraine and psychiatric comorbidities.

Cortical disexcitability in migraine, likely caused by a disruption of thalamocortical circuits (16), seems to be a relevant target for treatment. Indeed, in episodic migraine (EM) patients, a pattern of hyperresponsivity to sensory stimulation is observed during the interictal period. Hyperresponsivity has two main features: an average low-amplitude initial response followed by a lack of physiological habituation (i.e., a potentiation) of subsequent responses to repeated stimuli. By contrast, in the same interictal phase, CM patients show a normal level of preactivation and a normal habituation pattern, indistinguishable from that observed in EM patients during migraine attacks (16). This led to the hypothesis that CM is a permanent ictal-like state or “never-ending attack” (17), independently of the presence of the attack itself. Medication overuse may probably have a facilitating effect on this enduring altered excitability state, sticking patients in a preictal state, when normal preactivation is combined with lacking habituation (18). Taking together these results, we considered CM, either with or without MOH, as a “hyperexcitability disorder.” The fact that hyperexcitability is a common feature of the visual cortex in CM, with or without MOH, as well as in EM during the attack, has been highlighted by recent studies on flash-sound multisensorial perception (19, 20) and has been found to be greater in MOH patients with triptan abuse (18, 19).

The theory of cortical disexcitability in migraine provided a model for the development of different neuromodulation protocols. However, at present, there is no standard tDCS protocol for the treatment of migraine, as tDCS studies have yielded contrasting results. This inconsistency may be partially explained by the heterogeneity of the montages applied (21–26). Another relevant factor is the variability in the plastic and metaplastic effects of neuromodulation in migraine patients. Several studies showed that metaplastic changes in motor, auditory, or visual cortex induced by neurostimulation in healthy subjects (27–30) can be also found in migraine patients (31, 32), but they are influenced by the phase of the migrainous cycle in which neurostimulation is delivered (32). This additional source of variability may have affected the outcome of therapeutic trials with neuromodulation in the recent years.

Besides the occipital cortex, other brain areas have been also targeted by neurostimulation treatments for migraine. In a seminal repetitive transcranial magnetic stimulation trial in patients with CM, Brighina et al. found that excitatory stimulation of the left dorsolateral prefrontal cortex (lDLPFC) was associated with a better outcome compared to placebo (33), paving the road for subsequent noninvasive interventions. Unfortunately, the subsequent trials yielded contrasting results when lDLPFC was targeted (34, 35).

In recent years, the role of the right dorsolateral prefrontal cortex (rDLPFC) dysfunction has been highlighted in CM patients with psychiatric comorbidities and MOH (36, 37). Moreover, anodal stimulation of the rDLFPC in a dual montage is recommended as non-invasive neuromodulation therapy in addiction-related craving, a condition that could be similar to that of CM patients with MOH who are withdrawing acute medication for migraine (38, 39).

Considering the role of the visual cortex in the physiopathology of migraine and of the rDLPFC in medication overuse and psychiatric comorbidities in CM patients, in the present study we decided to pair occipital cathodal stimulation with anodal stimulation on the rDLPFC. We designed a pilot study recruiting difficult-to-treat patients who failed more than three previous preventive lines of therapy and/or had a relapse in analgesic overuse, despite several withdrawal attempts. These patients are at higher risk of comorbidities, including psychiatric disorders, and show a higher rate of failure to any preventive therapy. Considering (2) the lack of previous studies using neuromodulation on these target patients and (3) the new montage used, we decided to use a proof-of-concept design with no sham stimulation to test tolerability and feasibility of such a protocol. Regarding the potential efficacy of the tDCS treatment, we evaluated the reduction of the migraine burden and medication overuse. A second objective was to differentiate the clinical responses according to the psychiatric comorbidity profile of each patient.

## Materials and Methods

### Subjects and Clinical Records

The study was conducted at the Headache Center of Policlinico Umberto I in Rome. We recruited all consecutive patients (aged 18–65 years) diagnosed with CM (ICHD-III), according to the ICHD-III, with or without MOH (ICHD-III 8.2). Inclusion criteria were as follows: failure of three or more preventive treatments and/or relapse of MOH despite three or more withdrawal attempts. Exclusion criteria were as follows: other major neurological diseases, familial or personal history of seizures, cardiac arrhythmia, implanted pacemaker or metallic hardware in the head or neck, or contraindication to receive tDCS. Migraine preventive treatment was allowed only if it was unmodified and ineffective for ≥4 months prior to the beginning of the stimulation. All the patients were naive to tDCS.

Patients were asked to fill a headache diary to record clinical headache data (including medication intake) during the 30-day-long baseline assessment (t_0_), the 30 days of tDCS treatment (t_30_), and the 30 days of follow-up period (t_60_). Patients' quality of life at t_0_ was assessed using the Migraine Disability Assessment (MIDAS) and Six-item Headache Impact Test (HIT-6) scale. Moreover, we collected data about the main risk factors for migraine chronification, as listed by May and Schulte (2). All patients were instructed on MOH risks, and acute medication therapy withdrawal was verbally recommended.

### Psychiatric Assessment

Patients were recruited irrespectively of their psychiatric status and underwent a psychiatric interview by a trained psychiatrist before tDCS. Specifically, they were examined by means of the Structured Clinical Interview for *Diagnostic and Statistical Manual of Mental Disorders, Fourth Edition* Axis I Disorders (SCID-I) (40), the Akiskal's Temps-A scale for affective temperaments (41), the Diagnostic Criteria for Psychosomatic Medicine (DCPR) (42), and the Young Mania Rating Scale (YMRS) (43). All scales were administered before and within 2 weeks after the treatment.

All subjects gave written informed consent. This study was approved by the Ethics Committee of Human Experimentation of Policlinico Umberto I University Hospital and conformed to the latest version of the Declaration of Helsinki.

### Stimulation

TDCS was performed using a programmable DC brain stimulator (E.M.S., Bologna, Italy) with two rubber electrodes (5 × 5 cm) in sponges soaked with 3% saline. The anode was placed over the right dorsolateral prefrontal cortex (DLPFC) centered on F4 in the 10–20 electroencephalography system, and the cathode was positioned on the occipital cortex centered on Oz. During each session, a current of 2 mA was delivered for 20 min, with a fade-in/fade-out period of 20 s to decrease discomfort. TDCS was applied for 3 days per week for 4 weeks in the same hour of the day for each patient. In order to minimize variability, stimulation was applied during the morning and early afternoon hours (9 a.m. to 2 p.m.). The three stimulation sessions per week were scheduled individually with every patient and maintained stable throughout the treatment period.

### Outcome Measures and Statistical Analysis

We assessed the feasibility and safety of our tDCS stimulation protocol measured as number of dropouts and number and severity of reported side effects. Regarding the potential efficacy of tDCS as preventive therapy, our primary endpoint was the reduction of total headache days. Secondary outcomes were the reduction of severe and moderate headache days, acute medication intake, and psychiatric symptoms changes, as measured by validated scales. As subanalysis, we compared primary and secondary outcome measures between relevant subgroups (e.g., BD vs. non-BD, anxious vs. non-anxious patients).

We used the Shapiro–Wilks test to assess the normality of the variables. Hence, we used parametric and non-parametric tests on the basis of variables distributions. Repeated-measures analysis of variance (ANOVA) was used to investigate the effect of tDCS both at group level and between subgroups. Time points (t_0_, t_30_, t_60_) represent the within-group effect (time) and subgroups effect between groups (group) (e.g., patients with BD vs. patients without BD). For subgroups analysis, *post hoc* comparison and Bonferroni correction were used. Variables measured only twice (at baseline and posttreatment, as tests for psychiatric assessment) were compared using Student *t* test for paired sample or Wilcoxon signed ranks test according to their distribution. Spearman test was used for the correlation analysis as variables were normally distributed. Results were considered significant at *p* < 0.05 and were given by mean ± standard deviation. Statistical calculations were carried out using R statistical package (version 3.5.3).

## Results

Sixteen CM patients (four males; mean age = 53.88 ± 6.93 years) were recruited. All the patients were diagnosed with migraine without aura. Ten of 16 had MOH. The most overused drugs were non-steroidal anti-inflammatory drugs (38%), followed by combination drugs (19%), whereas only one patient overused triptans alone. At the moment of the recruitment, only one patient was under pharmacological prophylactic treatment for migraine having the other patients spontaneously discontinued for reported lack of efficacy; the number and classes of previous attempted prophylactic treatments are listed in Table 1. Eleven of 16 had already attempted a preventive therapy with onabotulinumtoxin without benefit. Risk factors for migraine chronification are listed in order of prevalence in Table 2.

**Table 1:** Demographic and clinical information of the population of the study.

**Patient ID**	**Age (y)**	**Sex**	**MOH**	**Prophylactic treatment**	**No. of past treatments**	**Classes of past treatments used**
Patient 1	55	F	Yes	No	7	AEDs, TCA, Ca-A, GON-B, OnaB, B-blockers, vitamins
Patient 2	62	F	Yes	No	7	AEDs, TCA, Ca-A, GON-B, OnaB, vitamins
Patient 3	56	F	Yes	No	3	AEDs, TCA, Ca-A,
Patient 4	61	F	No	No	4	TCA, Ca-A, OnaB, B-blockers
Patient 5	54	F	No	Valproate	3	AEDs, TCA, OnaB,
Patient 6	49	F	Yes	No	3	Ca-A, GON-B, OnaB
Patient 7	39	F	No	No	3	TCA, Ca-A, vitamins
Patient 8	51	M	Yes	No	4	B-blockers, AEDs, Ca-A, GON-B
Patient 9	60	F	Yes	No	5	B-blockers, TCA, AEDs, OnaB
Patient 10	55	M	Yes	No	6	AEDs, TCA, Ca-A, GON-B, OnaB, B-blockers
Patient 11	55	F	Yes	No	6	AEDs, TCA, Ca-A, GON-B, OnaB, B-blockers
Patient 12	59	F	No	No	7	AEDs, TCA, Ca-A, GON-B, OnaB, B-blockers
Patient 13	38	F	No	No	3	TCA, GON-B, OnaB
Patient 14	59	M	Yes	No	3	TCA, Ca-A, GON-B
Patient 15	54	F	Yes	No	4	TCA, Ca-A, OnaB, B-blockers
Patient 16	55	M	No	No	3	TCA, Ca-A, GON-B

*AEDs, antiepileptic drugs; B-blockers, β-blockers; Ca-A, calcium antagonists; F, female; GON-B, greater occipital nerve block; M, male; OnaB, onabotulinumtoxinB; TCA, tricyclin antidepressants*.

**Table 2:** Prevalence of factors associated with an increased risk for migraine chronification expressed as the number of patients and percentage of the sample.

**Risk factors**	**No**.	**%**
Female sex	12	75
Elevated body mass index	11	69
Allergy	8	50
Hypercholesterolemia	8	50
Analgesic drugs overuse	6	38
Sinusitis	5	31
Allodynia	4	25
Hypertension	4	25
Combination drugs overuse	3	19
Asthma	3	19
Bronchitis	3	19
Ictus or other cerebrovascular events	2	13
Ulcers	2	13
Triptan overuse	1	6
Emphysema	1	6
Cardiocirculatory diseases	1	6
Heart diseases	1	6
Arthritis	1	6

At baseline (t_0_), mean headache days were 20.50 ± 5.13. Severe headache days were 6.13 ± 5.95. MIDAS and HIT-6 mean scores were 81.38 ± 73.88 and 63.31 ± 4.55, respectively, indicating the highest level of disability associated with headache.

The SCID-I identified five patients affected by BD, whereas Akiskal's Temps-A scale for affective temperaments revealed six more patients within the bipolar spectrum, presenting cyclothymic or hyperthymic temperament. Overall, 11 (69%) of 16 patients displayed clinical or subthreshold presentations within the bipolar spectrum. SCID-I results showed that eight patients (50%) met the criteria for an anxiety disorder, two (12.5%) for a depressive disorder, and five (31%) for a somatoform disorder. Only four patients (25%) did not meet the criteria for any psychiatric comorbidity. Using DCPR, nine patients (56%) were found positive for type A, a behavioral pattern characterized by a strong competitivity, aggression, and hypervigilance.

### Safety and Efficacy of tDCS as Preventive Treatment

**Figure 1:**
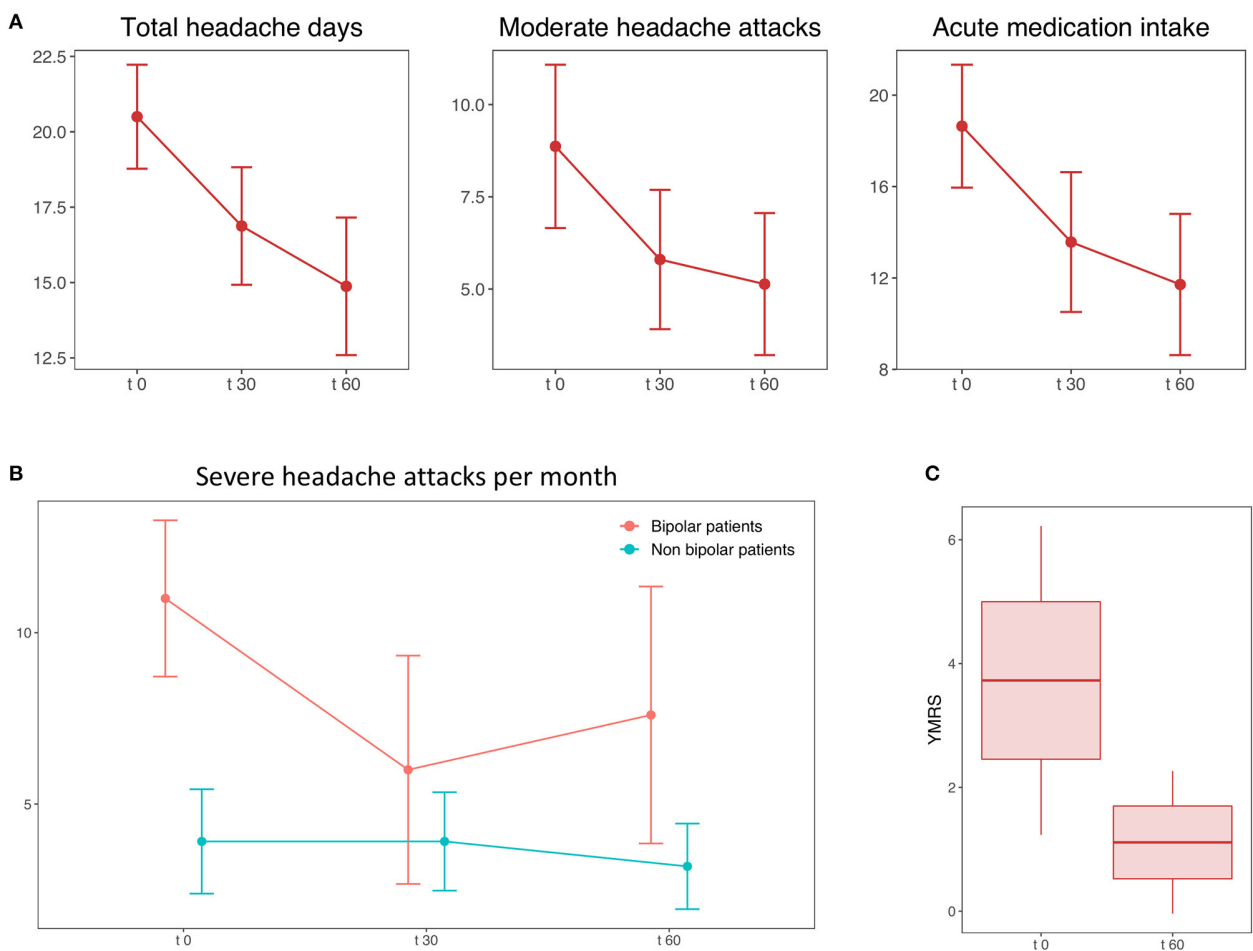
**(A)** The figure shows the mean and standard error of the number of total headache days (left plot), moderate attacks (central plot), and acute medication intake per month (right plot) at the baseline (t_0_), during the treatment (t_30_), and the follow-up period (t_60_). **(B)** Mean and standard error of the number of severe headache attacks at baseline (t_0_), during the treatment (t_30_), and the follow-up period (t_60_) in patients with (red line) and without (green line) bipolar disorder. **(C)** Significant decrease of Young Mania Rating Scale (YMRS) score before and after the treatment. The boxplot shows the mean, standard error and 90% CI of the score.

None of the 16 patients dropped out during the treatment period. No relevant adverse events were reported. In total, seven of 16 patients (44%) showed an improvement after tDCS. Repeated-measures ANOVA on total headache days showed a main effect of time: *F*_(2, 30)_ = 7.03, *p* = 0.012. *Post hoc* analysis revealed a significant reduction of total headache days from 20.5 ± 6.89 at baseline to 16.87 ± 7.8 at t_30_ (*t* = 2.45, *p* = 0.027) and to 14.81 ± 9.13 at t_60_ (−27% compared to baseline, *t* = 2.96, *p* = 0.01) (Figure 1). A main effect of time [*F*_(2, 26)_ = 7.07, *p* = 0.012] was also found for acute medication intake, which decreased from 18.64 ± 10.76 at baseline to 13.57 ± 12.24 at t_30_ (*t* = 2.51, *p* = 0.026) and to 11.71 ± 12.36 at t_60_ (−35% compared to baseline, *t* = 0.007, *p* = 0.02) (Figure 1A). Seven of 10 patients recovered from MOH. The reduction of severe headache days from 6.13 ± 5.95 at baseline to 4.56 ± 5.88 at t_60_ [−26%, main effect of time: *F*_(2, 30)_ = 2.57, *p* = 0.36] was not significant, whereas moderate headache attacks decreased [main effect of time: *F*_(2, 28)_ = 5.46, *p* = 0.04] from 8.87 ± 8.85 at baseline to 5.8 ± 7.55 at t_30_ (*t* = 2.39, *p* = 0.03) and to 5.13 ± 7.70 at t_60_ (*t* = 2.48, *p* = 0.027 compared to baseline) (Figure 1). Two patients complained a worsening of migraine after the stimulation.

### Subgroups Analysis

**Figure 2:**
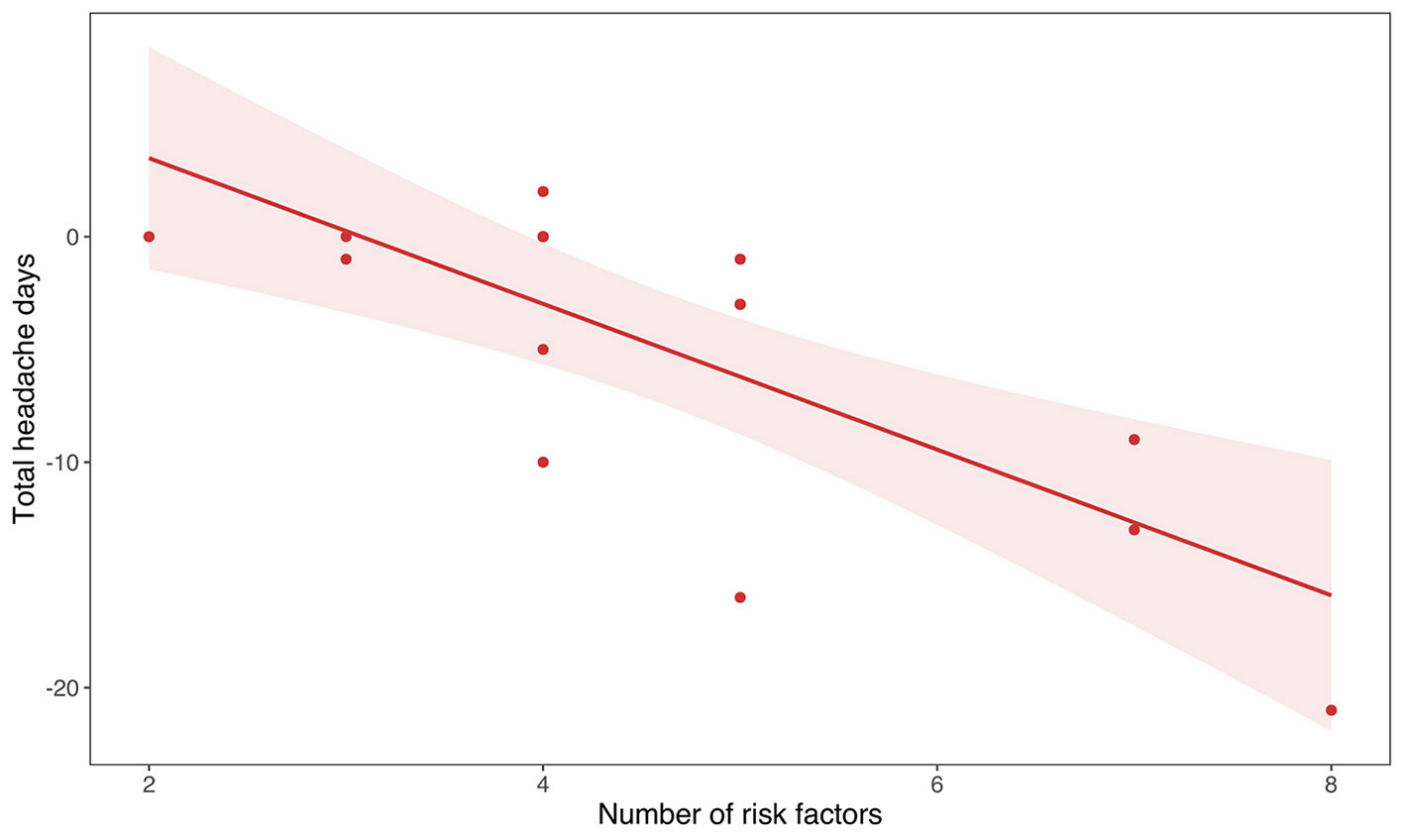
The graph shows the correlation between the total number of risk factors in each subject as listed in Table 1 and the reduction of the total number of headache days before and after the treatment (*R* = −0.7, *p* = 0.002).

Among all the psychiatric comorbidities observed, only the presence of clinical or subclinical forms of BD seemed to influence the outcome of the treatment. A repeated-measures ANOVA on the number of severe headache attacks found a significant interaction of time and group (patients with and without BD) [*F*_(2, 28)_ = 5.53, *p* = 0.036] (Figure 2). In details, patients with comorbid BD had a higher number of severe headache days/month at baseline (11.16 ± 4.68 vs. 3.1 ± 4.51; *t* = 2.59, *p* = 0.02), but showed a better response to tDCS compared to patients without BD (−3.4 ± 4.03 at t_30_ vs. −0.73 ± 3.03; −36% vs. −19%) with a significant reduction of severe headache days after 1 month of treatment (t_0_ vs. t_60_: *t* = 4.08, *p* = 0.01). No other significant result was found comparing the other subgroups.

After 1 month of tDCS, bipolar manic symptoms measured by YMRS in patients within the bipolar spectrum decreased from 4.55 ± 4.24 to 1.11 ± 1.76 (*t* = 2.97, *p* = 0.017) (Figure 1C). The number of risk factors for chronicity did not differ between patients with BD (*t* = 1.06, *p* = 0.3) and within the bipolar spectrum (*t* = 5.9, *p* = 0.557) and those who were not. The number of risk factors for migraine chronification was positively correlated with the reduction of total headache days (Spearman ρ = −0.7, *p* = 0.002) (Figure 1B). Allodynia had no impact on migraine at baseline (Spearman ρ = 0.36, *p* = 0.166) and the clinical response to tDCS (Spearman ρ = 0.32, *p* = 0.22).

## Discussion

In this proof-of-concept study, we demonstrated the feasibility and safety of a tDCS protocol based on the combination of cathodal stimulation over the visual cortex and anodal stimulation of the rDLPFC for migraine treatment. Moreover, we found that this protocol was effective as preventive therapy in difficult-to-treat CM, reducing migraine burden and medication intake. As CM is characterized by cortical hypersensitivity and an ictal-like habituation pattern (16), especially in the visual cortex (44, 45), we applied an inhibitory stimulation on the occiput to reduce the hyperexcitable state of the visual cortex. Moreover, inhibitory stimulation of the visual cortex was found effective in modifying the nociception at the brainstem level (46).

We decided to pair occipital cathodal stimulation with anodal stimulation of the rDLPFC. The choice of targeting the rDLPFC was based on its role in medication overuse and psychiatric diseases often comorbid with migraine, such as BD and depression (37, 47). Previous studies have shown that the reversion of DLPFC hypoactivity in BD is achieved after either pharmacological or psychotherapeutic successful treatment of BD symptoms (48). Moreover, rDLPFC dysfunction seems to be directly related to the intensity of psychiatric symptoms in migraine (37). Additionally, anodal tDCS on rDLPFC is effective in treating craving in drug abusers (15). Chronic migraineurs with MOH, which are the majority in our sample, have cerebral abnormalities in common with patients suffering from substance addiction (36, 38). This can also explain the reduction in analgesic use observed in our patients after tDCS.

To the best of our knowledge, this is the first study aiming to investigate the relationship between psychiatric comorbidities and the effect of tDCS treatment in CM patients. In our sample, we found a 69% prevalence of patients within the bipolar spectrum. According to the present literature, BD prevalence in migraineurs is approximately 19%, whereas in the general population the prevalence of BD is 2–4% (49). Other studies found a higher prevalence of migraine among patients with milder BD type II (34.8–70%) compared to those affected by the severe BD type I (19%) (12, 50).

We showed that patients with CM and BD had a heavier migraine burden at baseline. On the other hand, patients with CM and BD showed a better response to tDCS. The reduction of the migraine burden was paralleled by a more general improvement in psychiatric symptoms in patients within the bipolar spectrum, as measured by YMRS. A possible explanation is that, in addition to the direct effect on visual cortex hyperexcitability, our stimulation might have influenced also other brain areas involved in both CM and BD. Apart from local activity, tDCS is also able to durably induce long-range modulation of brain cortex excitability (8), having complex and pleiotropic effects on brain activity. In particular, occipital stimulation has been previously used to target the brainstem and the cerebellum (46).

The prefrontal–thalamic–cerebellar circuit is involved in cognitive, sleep, and social impairment in BD (51, 52). Accordingly, prefrontal–occipital tDCS stimulation improved sleep quality, visuospatial memory, and executive functions in euthymic BD patients (53, 54). Interestingly, cerebellum is also involved in the processing of nociceptive stimuli (55–57). Therefore, incidental modulation of cerebellar activity by means of occipital tDCS might account for the parallel improvement in BD and migraine symptoms in our sample.

Finally, we highlighted a correlation between the number of risk factors and the clinical benefit of the patients. One possible explanation is that some risk factors (e.g., asthma, arterial hypertension, and elevated body mass index), or risk related to the pharmacological therapy of comorbidities, might have prevented patients from receiving some lines of treatment (e.g., β-blockers or valproate) or have caused a precocious discontinuation. For this reason, in patients with a high number of risk factors, tDCS could be effectively used because of the absence of side effects or drug interactions.

However, some limitations of this study are worth to be mentioned. First, a major limitation is that a placebo effect cannot be ruled out, and the current results must be confirmed by a randomized sham-controlled trial. Estimating the placebo effect in clinical trials for migraine treatment is not straightforward, as it may vary according to the complexity of the procedure (58). Our study tested the effect of a new montage on a pool of difficult-to-treat patients (on average 4.4 lines of treatment per patients, with 11 patients who had previously attempted a preventive therapy with onabotulinumtoxin without any benefit) reporting good tolerability and no dropouts. Moreover, 44% of the patients experienced an improvement, and seven patients reverted from MOH, encouraging the use of these preliminary data to calculate the appropriate sample size for a randomized controlled trial.

The lack of previous studies using this type of montage prevented us from predetermining our own sample size on a statistical basis, which is a second limitation to the current pilot study. The sample size was simply limited by the availability of patients matching the inclusion criteria for the study in our headache center. However, the final sample size is in line with the recommendations for pilot studies (59). To minimize the bias in evaluating the efficacy of the treatment, we planned a primary outcome—i.e., testing prefrontal–occipital tDCS efficacy on the basis of total and severe days of headache—and a limited number of exploratory analyses, to keep the number of statistical tests to a minimum. Moreover, we applied Bonferroni correction, which is the most conservative correction in *post hoc* comparisons, to minimize the type I error (60).

In third place, the lack of a complete neurophysiological evaluation prevented us from directly assessing the effect of tDCS at the neural level. The inclusion of neurophysiological parameters in future studies would allow to evaluate the efficacy of the tDCS treatment and to compare different tDCS protocols on more solid bases.

Indeed, plastic changes induced by neurostimulation can vary according to the phases of the migraine cycle (32). Therefore, there is the possibility that the stimulation may act differently depending on the frequency of the migraine attacks (i.e., CM vs. EM patients). In order to minimize the effect of such variability, we only included patients with CM in our sample, with an average number of 20.5 days of headache days per month.

Moreover, without a direct neurophysiological read-out, we could only speculate about the brain networks affected by the stimulation. In particular, we could not directly assess the neural effects of stimulating the right rather than the left DLPFC, or the lack of lateralization in the case of the occipital cortex. Indeed, the use of a midline cathodal stimulation over the visual cortex does not allow disentangling between a lateralized effect on the activation of one hemisphere and the presence of interhemispheric interactions. However, while an impairment of the interhemispheric inhibition at the level of the sensory–motor cortex has been recently described in migraine, this seems to be a feature of migraine with aura, which was not present in our sample (61).

## Conclusions

In our proof-of-concept study, anodal rDLPFC and cathodal occipital tDCS was found to be safe and effective in reducing both the number of total headache days and acute medication intake per month in difficult-to-treat CM patients. Moreover, we found that, in spite of a worse clinical status at baseline, patients with comorbid BD had a better response to tDCS with respect to both headache and psychiatric symptoms. Although in most of clinical trials on migraine prevention using central non-invasive neurostimulation patients affected by psychiatric comorbidities are excluded, in this proof-of-concept study we aimed to investigate the impact of psychiatric comorbidities on the outcome of tDCS treatment in CM patients. If on one hand, this choice makes it harder to assess whether the stimulation acts directly on migraine pathophysiology or not, on the other hand it is a pragmatic approach to be implemented in difficult-to-treat CM patients, in which psychiatric comorbidities are often present.

## Data Availability Statement

The datasets presented in this article are not readily available because access is subject to approval by the Policlinico Umberto I Ethics Committee. Requests to access the datasets should be directed to giuliomastria@msn.com.

## Ethics Statement

The studies involving human participants were reviewed and approved by Policlinico Umberto I Ethics Commitee. The patients/participants provided their written informed consent to participate in this study.

## Author Contributions

GM, AV, VM, RD, and VD contributed to the design of the study. GM, AC, VM, CP, and SB collected the data. GM, AV, and VM analyzed the data and wrote the first draft of the paper. All the authors revised the paper for intellectual content.

## Conflict of Interest

The authors declare that the research was conducted in the absence of any commercial or financial relationships that could be construed as a potential conflict of interest.
